# Prenatal diagnosis and molecular cytogenetic characterization of 12 cases of chromosome 8 inverted duplication deletion syndrome

**DOI:** 10.1186/s13023-025-03969-w

**Published:** 2025-08-11

**Authors:** Xi Yang, Rong Hu, Weiwei Huang, Jian Lu

**Affiliations:** 1 Guangdong Provincial Research Center for Sports Assistive Device Design Engineering and Technology, Guangzhou Sport University, No.1268, Guangzhou Avenue Middle, Tianhe District, Guangzhou, 510500 Guangdong People’s Republic of China; 2 Medical Genetic Center, Guangdong Women and Children Hospital, NO.521-523, Xingnan Road, Panyu District, Guangzhou, Guangdong, 511442 People’s Republic of China

**Keywords:** 8p inverted duplication deletion, Karyotype analysis, Chromosomal microarray analysis, Congenital heart disease, Abnormal cerebral structure, Craniofacial dysmorphism, Olfactory receptor gene clusters

## Abstract

**Background:**

Inverted duplication of the short arm of chromosome 8 (inv dup del (8p)) and the deletion of its adjacent terminal represent a rare chromosomal rearrangement. To date, only a limited number of prenatal cases have been documented from a molecular cytogenetic perspective. This study investigates the molecular genetic characteristics and intrauterine ultrasound phenotypes of fetuses prenatally diagnosed with inv dup del (8p).

**Methods:**

We retrospectively analyzed chromosomal microarray analysis (CMA) results from cases seeking prenatal diagnosis at the Medical Genetics Center of Guangdong Women and Children’s Hospital from January 2016 to December 2022. We identified 12 prenatal cases of inv dup del (8p) and summarized their prenatal clinical manifestations and associated genes by combining ultrasound findings with literature review.

**Results:**

Both G-banding and CMA techniques confirmed the presence of interstitial duplication with concomitant terminal deletion of chromosome 8’s short arm in all 12 cases. The locations and lengths of the 8p duplications varied in their proximal breakpoint. Observed ultrasound findings included fetal increased nuchal translucency (NT), lateral cerebral ventricular dilatation, craniofacial dysmorphisms and abnormalities of the brain, heart and kidneys. Ectopic recombination appears to be the dominant mechanism for rearrangement formation in cases 1–11. In contrast, case 12 exhibited inv dup del (8p) without an intact region between duplication and deletion, which is better explained by the U-type exchange mechanism.

**Conclusion:**

The intrauterine phenotypes of inv dup del (8p) are diverse, with cerebral and cardiac anomalies being the most commonly observed ultrasound findings. However, these clinical manifestations are not specific to inv dup del (8p), and some fetuses may not exhibit noticeable ultrasound abnormalities during early gestation. Therefore, definitive diagnostic testing through karyotyping and CMA is essential. Additionally, CMA enables precise detection of copy number variations (CNVs), including exact size and genomic location. This detailed information is critical for accurate genetic counselling and helps clarify the mechanism behind the inv dup (8p) rearrangement.

**Supplementary Information:**

The online version contains supplementary material available at 10.1186/s13023-025-03969-w.

## Introduction

Inverted duplication deletion of the short arm of chromosome 8 (inv dup del (8p)) is a rare and complex chromosomal rearrangement with an estimated frequency of 1/10,000–1/30,000 in liveborn infants [1, 2]. This rearrangement involves a deletion of the telomeric region (8p23.1-pter) and a concomitant inverted duplication from 8p23.1 to the centromere region of variable size [3–6]. The proximal breakpoints of the duplicated region are often located between 8p11.2 and 8p12, while distal breakpoints are frequently found in the 8p22 or 8p23.1 regions [1–15]. The syndrome is characterized by a wide range of clinical features, including intellectual disability, craniofacial dysmorphisms, central nervous system (CNS) anomalies, congenital heart defects, and hypotonia [1–4, 6–15]. The severity of clinical manifestations is often correlated with the size of the duplicated region, although exceptions have been noted [2–4]. Inv dup del(8p) typically results from abnormal meiotic recombination events, such as ectopic recombination and U-type exchanges [4, 11]. It is considered that a disomic spacer region (4.9–5.5 Mb) is characteristic of ectopic recombination events. If recombination occurs between homologous sequences in non-homologous genomic regions, it is classified as ectopic recombination [5]. However, in some cases, no neutral copy region is present, suggesting the formation of inv dup del (8p) through U-type exchange mechanisms [4]. These recombination processes are closely associated with low-copy repeats (LCRs). LCRs with high homology play a key role in various genomic rearrangements. On chromosome 8p23.1, there are two such LCRs: the repeat distal (REPD) and the repeat proximal (REPP). Each contains several olfactory receptor genes and defensin genes, which may have a causal relationship with the inverted duplication of chromosome 8 (inv dup (8p)) [1–6].

To date, over 100 clinically diagnosed postnatal cases of inv dup del (8p) have been published since the first case was reported by Weleber et al. in 1976 [3–5, 14, 16]. However, the literature concerning the prenatal detection of this genetic disorder remains limited. Thus far, only a few prenatal cases of inv dup del (8p) have been reported [17–23]. The previously published prenatal cases indicate that the fetal karyotype typically shows terminal deletion (del(8)(p23.1-pter)) and inverted duplication (inv dup (8p)) of chromosome 8p, with the specific range varying among individuals. In certain cases, mosaicism may be observed, wherein distinct cell lines exhibit different chromosomal rearrangements (i.e., del (8p) in some cells and inv dup (8p) in others) [18]– [19, 22]. The phenotypic manifestations of fetuses with inv dup del (8p) syndrome are diverse and primarily include neurological abnormalities (e.g., hydrocephalus, cerebellar hypoplasia, agenesis or dysplasia of the corpus callosum, ventriculomegaly, and intellectual disability), congenital heart disease (CHD) (e.g., ventricular septal defect, polyvalvular dysplasia, dilated pulmonary arteries and right ventricular hypertrophy), hydronephrosis, dysmorphic facial features (e.g., prominent forehead, micrognathia, large low-set ears, hypertelorism and wide nasal base) and limb deformities (e.g., hand and foot deformities, limb contractures and clubfeet) [17–23]. Additional ultrasound findings include polyhydramnios, oligohydramnios, fetal hydrops and intrauterine growth restriction [17–20]. Most studies suggest that these rearrangements originate from the mother, possibly due to abnormal recombination events during maternal meiosis [17]– [18, 22]. Overall, the prenatal diagnosis of inv dup del(8p) syndrome currently faces several challenges, including diagnostic complexity, phenotypic heterogeneity, and the complexity of predicting clinical outcomes based on genetic findings. In the early gestation, ultrasound may fail to detect subtle anomalies. However, potential chromosomal abnormalities in a fetus may lead to abnormal development both prenatally and postnatally, increasing the difficulty of prenatal counseling. Severe intracranial and cardiac anomalies are often detected only in later stages of gestation or postnatally [17, 21]. Additionally, confined placental mosaicism (CPM) may result in discordant chromosomal abnormalities between placental and fetal tissues, further complicating prenatal diagnosis [18]– [19]. In this study, to further understand the characteristics and genetic mechanisms of inv dup del (8p), we report 12 prenatal cases of this syndrome through a retrospective analysis of all cases tha*t* underwent prenatal chromosomal microarray analysis (CMA) at Guangdong Women and Children Hospital from January 2016 to December 2022. We also review the clinical manifestation and molecular cytogenetic features of this syndrome. Our objective is to analyze the fetal ultrasound findings in these cases to ascertain whether there are any characteristic features of inv dup del (8p). If such features can be identified, they will be beneficial to the prenatal diagnosis of inv dup del (8p). By synthesizing the results of chromosomal karyotype analysis and CMA of these cases, we can better understand the chromosomal rearrangements and the relationship between gene deletions/duplications and clinical manifestations, thereby enhancing the capability of genetic counseling.

## Materials and methods

### Subjects

We conducted a retrospective review of CMA results for prenatal diagnosis cases at the Medical Genetics Center of Guangdong Women and Children’s Hospital from January 2016 to December 2022. During this period, a total 103,840 cases underwent CMA and karyotype analysis. These samples comprised 5,023 chorionic villus samples, 9,0547 amniotic fluid samples, and 8,270 umbilical cord blood samples. We identified 12 cases of inverted duplication deletion of the short arm of chromosome 8 (inv dup del (8p)). Clinical data of all prenatal cases that received CMA were carefully collected. The patients originated from 47 medical institutions in Guangdong province. Each case underwent routine ultrasound and serological screening/non-invasive prenatal screening (NIPS) at a primary hospital; if any of the screening results were abnormal, the case was referred to the Medical Genetic Centre for CMA. Chorionic villus sampling was performed at 11–14 weeks of gestation. Amniocentesis and cordocentesis were conducted to collect amniotic fluid from patients at 17–24 weeks of gestation and cord blood from those with gestational age over 24 weeks. This study has been approved by the Institutional Review Board/ Medical Ethics Committee of Guangdong Women and Children Hospital (IRB reference number: 202401297). All patients provided a written informed consent.

### Chromosomal G-banding analysis

Chorionic villi, amniotic fluid and fetal cord blood were collected by villus sampling, amniocentesis and cordocentesis, respectively, after obtaining signed informed consent from the patients. Metaphase chromosome G-banding (320–400 bands) karyotyping was performed in accordance with standard protocols [24]. Karyotypes were described following the criteria set forth by the International System for Human Cytogenetic Nomenclature 2020 (ISCN 2020).

### Chromosomal microarray analysis

Fetal uncultured genomic DNA was isolated from the chorionic villi, amniotic fluid and cord blood samples using the QIAamp DNA Mini Kit (QIAGEN, Germany) following the manufacturer’s protocols. The isolated DNA was quantified with a NanoDrop 2000 spectrophotometer (Thermo Fisher Scientific, USA). Only DNA samples with a concentration greater than 20ng/µL and optical densities reaching 1.8 to 2.0 at 260/280 nm were selected for further analysis and subsequently stored at -20℃. SNP-array analysis was performed using the CytoScan 750k array (Thermo Fisher Scientific, USA), which features 550,000 copy number variations (CNVs) markers and 200,000 SNP markers, providing an average resolution of 100 kb. Data analysis was conducted using Chromosome Analysis Suite software (ChAS v.4.2, Thermo Fisher Scientific, USA). Databases (DECIPHER, OMIM, ClinGen, DGV, etc.), relevant literature and guidelines were used for the interpretation of CMA results.

## Results

**Fig. 1:**
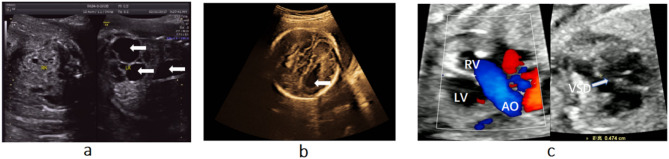
The detailed ultrasonography showing the abnormal findings in cases 1, 4 and 12, with arrows pointing to the abnormal parts. (**a**) Multiple anechoic areas are seen in the left kidney of case 1, suggesting polycystic kidney disease. (**b**) A teardrop-shaped change is observed in the lateral ventricle of case 4, indicating a possible absence of the corpus callosum. (**c**) In case 12, the right ventricle (RV) is connected to the aorta (AO), suggesting double outlet right ventricle, and a ventricular septal defect measuring 0.474 cm is present

Between January 2016 and December 2022, 12 cases of inv dup del (8p) were identified among 103,840 patients who underwent invasive prenatal diagnosis through CMA in our clinic. The average age of the 12 pregnant women was 29.2 years old (range: 24–39). Of these cases, eleven chose voluntary termination of pregnancy, while one case (case 11) continued the pregnancy and delivered a female infant in another hospital. Follow-up at five years of age showed no apparent abnormalities, and she is attending a regular kindergarten. Her father’s CMA result is consistent with hers. His highest level of education is high school and he is engaged in sales-related work. The clinical and genetic characteristics of the 12 cases are shown in Table 1. Figure 1 shows ultrasound imaging abnormalities observed in selected cases. In Fig. 1a, a polycystic kidney is noted in case 1; In Fig. 1b, the absence of the corpus callosum is evident in case 4; In Fig. 1c, case 12 presents with a ventricular septal defect. Additionally, the right ventricle (RV) is connected to the aorta(AO). This suggests a double outlet right ventricle.

**Fig. 2:**
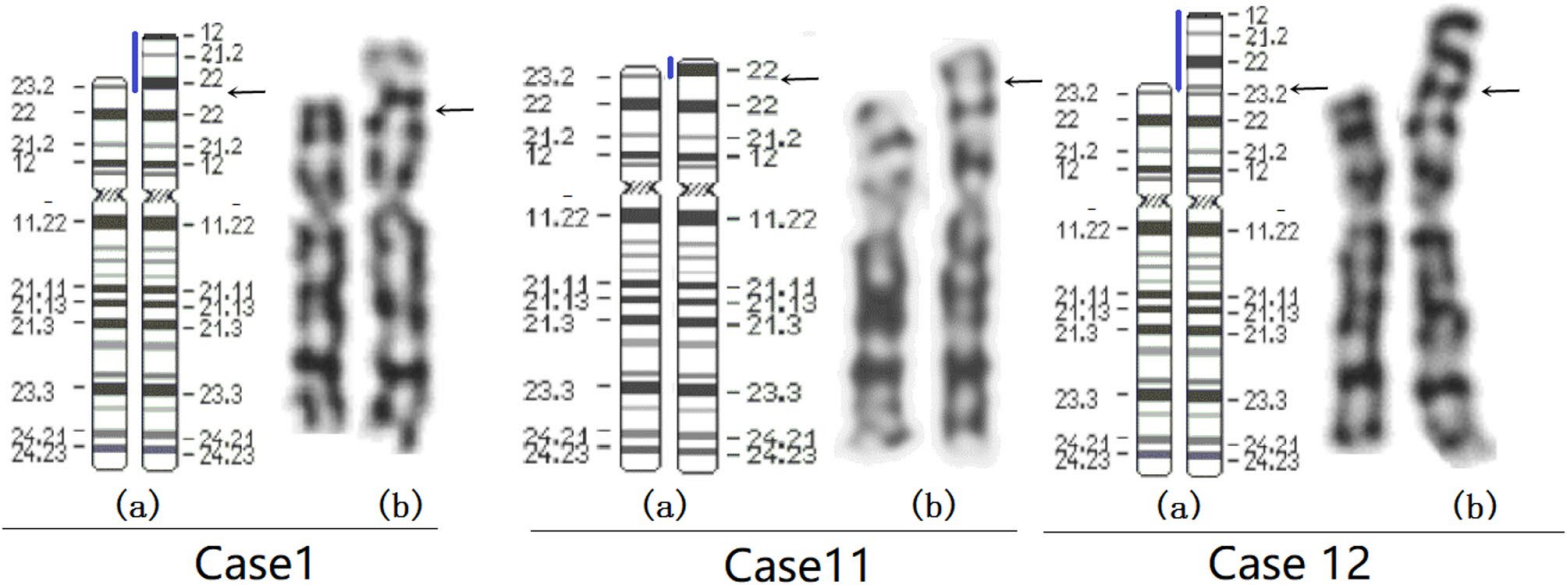
Karyotype diagrams of Case 1, 11 and 12. (**a**) ideograms of normal chromosome 8 (left) and rearranged chromosome 8 (right). (**b**) G-banded karyotypes of normal chromosome 8 (left) and inv dup del (8p) (right).The black arrows indicate the positions of chromosomal rearrangement breakpoints, and the blue lines represent the duplicated regions

**Fig. 3:**
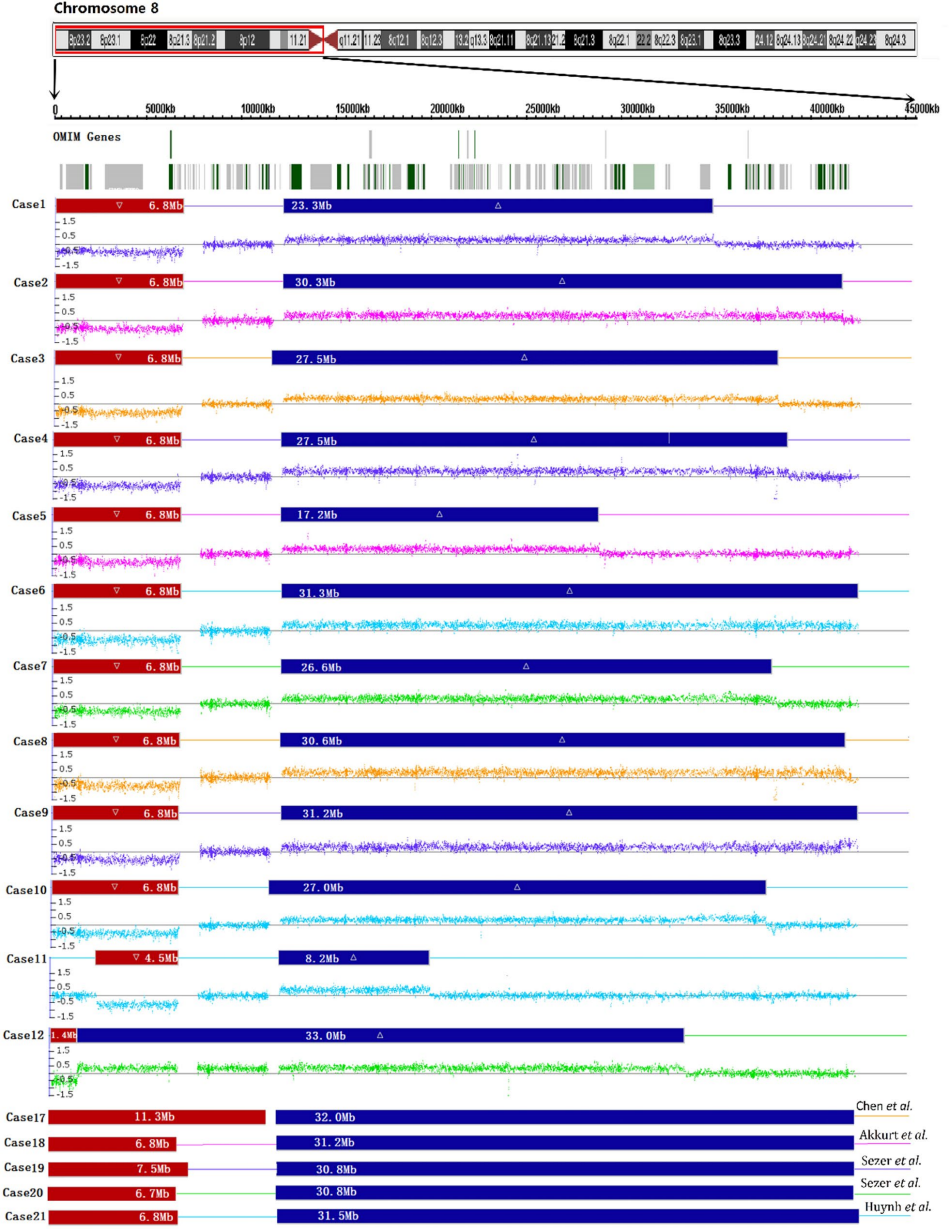
CNVs and hybridization profiles in the short arm of chromosome 8 (8p) in 17 cases with inv dup del (8p) by CMA. Cases 1–12: Present study; Cases 17–21: Previous studies; Cases 13–16 were analyzed using karyotyping and are not applicable to this figure; Red boxes: deletion; Blue boxes: duplication; The line between deletion and duplication represents the 8p23.1 region

Chromosome G-banding results revealed an inverted duplication in the chromosome 8p in all 12 cases (Table 1). For example, the karyotype for case 1, 11 and 12 involving chromosome 8 are shown in Fig. 2. The CNVs and hybridization profiles of the chromosome 8p, as revealed by CMA, are detailed in Table 1; Fig. 3. CMA confirmed the presence of duplications and terminal deletions in the chromosome 8p in all 12 cases. Cases 1–11 showed a deletion of 4.5–6.8 Mb at the distal end of chromosome 8p23, followed by a neutral copy region of 4.9–5.5 Mb, and subsequently a duplicated region of 8.2–31.3 Mb. Notably, case 11 had an 8.2 Mb duplicated region, which was the smallest among the 12 cases, while the duplicated regions in the remaining cases ranged from 17.2 to 33 Mb (Table 1). Cases 1–10 showed a typical inv dup del (8p) rearrangement with a 6.8 Mb deletion, whereas case 11 had a 4.5 Mb deletion. The breakpoints of the 8p deletions in cases 1–11 were consistently mapped to the REPD region of 8p23.1, while the distal breakpoints of the 8p duplications corresponded to the REPP region of 8p23.1. In contrast, case 12 presented with a 1.8 Mb deletion, immediately followed by a 33.0 Mb duplication, without an intact region in between (Table 1; Fig. 3). CMA revealed only abnormalities in chromosome 8 in cases 1–12.

The literature concerning the prenatal diagnosis of inv dup del (8p) is limited. MacMillin et al. first reported two prenatal cases of inv dup del (8p) in 2000 [17]. To date, only a few prenatal cases of inv dup del (8p) have been reported in previous studies [17–23]. In Table 1, we summarized clinical manifestations, karyotype and CMA results, sizes of deletions/duplications and gene numbers for patients in this study and previous reports. Additionally, we compared our CMA results with those of five previous cases, including chromosomal locations and the sizes of deletions/duplications.

**Table 1:** Clinical features summary of patients identified by karyotype analysis/cma (Present & previous Studies)

Case No.	References	Maternal age (years)	Gestation (week)	Sample	Clinical features/ Indications for prenatal diagnosis	Karyotype	CMA	Segment size(Mb)(Number of OMIM genes)	Follow up	Parental study
1	N/A	26	27	CB	Left cerebral ventricular dilatation, polycystic left kidney, echoless area of right kidney	46,XY, der(8)del (8)(p23)dup(8)(p23p12)	8p23.3p23.1(158,048 − 7,044,046)x1 8p23.1p12(12,532,885 − 35,826,545)x3	Deletion 6.8 Mb(17) Duplication 23.3 Mb(121)	Terminated	*De novo*
2	N/A	29	18	AF	History of two spontaneous abortions at the 2nd week of pregnancy	46,XY, der(8)del (8)(p23)dup(8)(p23p11.2)	8p23.3p23.1(158,048 − 7,044,046)x1 8p23.1p11.21(12,532,773 − 42,869,979)x3	Deletion6.8 Mb(17) Duplication30.3 Mb(169)	Terminated	N/A
3	N/A	28	34	CB	Left lateral cerebral ventricular dilatation	46,XX, der(8)del (8)(p23)dup(8)(p23p11.2)	8p23.3p23.1(158,048 − 6,999,114)x1 8p23.1p11.22(11,936,000–39,388,919)x3	Deletion 6.8 Mb(17) Duplication 27.5 Mb(148)	Terminated	*De novo*
4	N/A	33	24	AF	Bilateral hydrocephalus, agenesis of the corpus callosum	46,XX, der(8)del (8)(p23)dup(8)(p23p11.2)	8p23.3p23.1(158,048 − 7,044,046)x1 8p23.1p11.21(12,490,998 − 40,004,763)x3	Deletion6.8 Mb(17) Duplication 27.5 Mb(148)	Terminated	N/A
5	N/A	26	17	AF	Thick NT, echogenic intracardiac focus, abnormal serum screening result	46,XX, der(8)del (8)(p23)dup(8)(p23p12)	8p23.3p23.1(158,048 − 7,044,046)x1 8p23.1p12(12,532,773 − 29,764,500)x3	Deletion 6.8 Mb(17) Duplication17.2 Mb(102)	Terminated	N/A
6	N/A	27	27	AF	Cerebellar vermis hypoplasia	46,XY, der(8)del (8)(p23)dup(8)(p23p11.1)	8p23.3p23.1(158,049 − 7,044,046)x1 8p23.1p11.1(12,549,214 − 43,824,035)x3	Deletion 6.8 Mb(17) Duplication 31.3 Mb(173)	Terminated	N/A
7	N/A	30	30	CB	Cerebellar vermis hypoplasia	46,XX, der(8)del (8)(p23)dup(8)(p23p11.2)	8p23.3p23.1(158,049 − 7,044,046)x1 8p23.1p11.22(12,527,949 − 39,128,519)x3	Deletion 6.8 Mb(17) Duplication26.6 Mb(145)	Terminated	N/A
8	N/A	39	19	AF	Increased fetal cardio-thoracic ratio, fetal tricuspid regurgitation, persistent left superior vena cava, increased interocular distance, racquet-shaped placenta	46,XY, der(8)del (8)(p23)dup(8)(p23p11.2)	8p23.3p23.1(158049_6995855)x1 8p23.1p11.22(12490999_43123980)x3	Deletion6.8 Mb(17) Duplication30.6 Mb(172)	Terminated	N/A
9	N/A	32	18	AF	Abnormal serum screening result	46,XX, der(8)del (8)(p23)dup(8)(p23p11.1)	8p23.3p23.1(158049_6959820)x1 8p23.1p11.1(12551618_43824035))x3	Deletion 6.8 Mb(17) Duplication 31.2 Mb(173)	Terminated	N/A
10	N/A	29	25	AF	Single umbilical artery, persistent omphalocele	46,XY, der(8)del (8)(p23)dup(8)(p23p11.2)	8p23.3p23.1(158049_6962251)x1 8p23.1p11.22(11936001_38901732)x3	Deletion 6.8 Mb(17) Duplication27.0 Mb(147)	Terminated	N/A
11	N/A	24	18	AF	Partial duplication of chromosome 8 indicated by non-invasive prenatal screening, left lateral cerebral ventricle slightly enlarged	46,XX, der(8)dup(8)(p23p21.3)	8p23.2p23.1(2,571,238-7,044,046)x1 8p23.1p21.3(12,532,773 − 20,684,233)x3	Deletion 4.5 Mb(10) Duplication8.2 Mb(27)	Delivered	pat
12	N/A	27	32	CB	Fetal double outlet right ventricle/double subarterial ventricular septal defect, hydropericardium, fetal biparietal diameter greater than gestational age, mild dilatation of the left lateral ventricle by 11 mm (MR).	46,XX, inv dup(8)(p23p12)	8p23.3(158,048 − 1,554,606)x1 8p23.3p12(1,561,791 − 34,523,224)x3	Deletion 1.4 Mb(5) Duplication33.0 Mb(174)	Terminated	*De novo*
13	Martha et al.,2000	29	16 + 5	AF	Severe bladder dilatation, bilateral hydronephrosis, abnormal lower lumbar spine, Dandy-Walker lesions (hemispherical cyst and enlarged third ventricle) and mild oligohydramnios	46,XY, del (8)(p23),inv dup (8)(p23p11.2)	N/A		N/A	*De novo*
14	Martha et al.,2000	34	30	AF	Mild lateral cerebral ventricular enlargement, agenesis of the corpus callosum, Dandy-Walker lesions, normal ultrasound findings at 16 and 18 gestational weeks	46,XX/XY, del(8)(p23),inv dup (8)(p23p11.2)	N/A	N/A	Delivered	*De novo*
15	Soler et al.,2003	39	12 + 4/15	CVS/AF	Club feet, clenched left hand, subcutaneous edema and bilateral hydrocephalus	CVS:46,XX, i(8q)[7]/46,XX, del(8)(p11.2)[28]. AF:46,XX, dup (8) (p23p11.2)[21]	N/A	N/A	Terminated	*De novo*
16	Pramparo et al.,2004	32	12	CVS	Subcutaneous edema in the neck, generalized fetal hydrops, severe echocardiographic abnormalities (atrial and ventricular septal defects, dilated left ventricle and pericardial effusion)	CVS direct:46,XX, del(8)(p11.2)[26] CVS culture:46,XX, inv dup(8p)[98]	N/A	N/A	Terminated	*De novo*
17	Chen et al.,2016	19	31	CB	Right ventriculomegaly, hypoplastic left heart, polyhydramnios and intestinal obstruction	46,XX, der (8)del (8) ((p23.1),inv dup (8)((p23.1p11.1)	8p23.3p23.1(191530-11536657)x1 8p23.1p11.1(11545953–43541986)x3	Deletion 11.3 Mb(51) Duplication 32.0 Mb(180)	Delivered	*De novo*
18	Akkurt et al.,2016	38	31	AF	Small fetus with polyhydramnios, absent nasal bone, clenched left hand, agenesis of the corpus callosum, cerebellar hypoplasia, dilated third cerebral ventricle, polyvalvular dyplasia and complex cardiovascular disorder	N/A	8p23.3p23.1(158048–6999114)x1 8p23.1p11.1(12552775–43780262)x3	Deletion 6.8 Mb(17) Duplication31.2 Mb(173)	Delivered	N/A
19	Sezer et al.,2018	39	12	CVS	Cerebellar hypoplasia, hemispheric septal cyst, ventricular septal defect and echocardiography at 18 gestational weeks, NT thickening at 12 gestational weeks	der (8)del (8) ((p23),inv dup (8)(p23p11.1)	8p23.3p23.1(191530–7691960)x1 8p23.1p11.1(12404003–43191315)x3	Deletion7.5 Mb(17) Duplication 30.8 Mb(173)	Terminated	*De novo*
20	Sezer et al.,2018	39	16	AF	Ventricular septal defect and asymmetric left and right ventricles at 19 gestational weeks	der (8)del (8) ((p23),inv dup (8)(p23p11.2)	8p23.3p23.1(191530–6880363)x1 8p23.1p11.21(12039930–42755506)x3	Deletion6.7 Mb(16) /Duplication30.8 Mb(171)	Terminated	*De novo*
21	Huynh et al.,2021	32	11 + 4	CVS	NT thickening, diffuse lymphedema	CVS direct: mos 46,XX, i(8)(q10) [13]/46,XX, del (8)(p23)[10] CVS culture:46,XX, der (8)del(8)(p23)dup(8)(p? )[18]	8p23.3p23.1(191530–6880363)x1, 8p23.1p11.1 (12039930–43529733)x3	Deletion 6.8 Mb(17) Duplication31.5 Mb(175)	Terminated	*De novo*
N/A: Not applicable, CB: Cord blood, AF: Amniotic fluid, NT: Nuchal translucency, CVS: Chorionic villus sample, pat: Paternal inheritance, Cases 1–12: Present study; 13–21: Previous studies										

## Discussion

The inv dup del (8p) is primarily caused by non-allelic homologous recombination (NAHR), a form of ectopic recombination that frequently occurs between low copy repeats (LCRs) known as REPD and REPP, which flank the 8p23.1 region [1, 6, 13, 25]. These two olfactory receptor gene clusters, REPD and REPP, exhibit complex DNA sequences featuring retroviral elements, olfactory receptor genes, and defensin genes [6, 26]. It has been reported that the breakpoints of inv dup del (8p) originate primarily from the maternal chromosome and lead to the deletion of the maternal chromosome. Fisch et al. further demonstrated the presence of heterozygous inversions in the two olfactory receptor regions on the maternal chromosome 8p23.1, with incidence rates of 26% and 27% in European and Japanese populations, respectively [10]. Polymorphic inversions and LCRs can induce chromosomal rearrangements, thereby increasing the incidence of inv dup del (8p) compared to other *de novo* chromosomal structural abnormalities [1, 26, 27].

Three mechanisms of chromosomal rearrangement induced by REPD and REPP are currently thought to be involved in the origin of inv dup del (8p) [4, 28–31]. The first mechanism involves one of the parents being a carrier of a paracentric inversion, leading to an exchange between non-sister chromatids of the two paired chromosomes by a crossing-over event within the inverted region [28]. This mechanism is characterized by the formation of a derivative chromosome with a neutral copy region between the duplicated and deleted segments. Conversely, the second mechanism is based on the folding, pairing and recombination between sister chromatids within a chromosome, similarly resulting in a neutral copy region between the duplicated and deleted segments [29]. The third mechanism depends on a premeiotic double-strand break in sister chromatids, followed by the fusion of the broken ends, leading to a U-type exchange between sister chromatids [30–32]. The first two mechanisms typically result in a neutral copy region between the duplicated and deleted regions on the derivative chromosome. In contrast, the third mechanism is distinct in that the duplicated region is directly adjacent to the deleted region, without an intervening copy region. In our study, cases 1–11 and 18–21 align with the first two mechanisms, as indicated by the presence of a neutral copy region between the duplicated and deleted regions. In contrast, case 12 and case 17 may be result from by the U-type exchange mechanism, as evidenced by the direct adjacency of the duplicated and deleted regions.

Inv dup del (8p) rearrangement presented a deletion at the distal end of chromosome 8p23. The breakpoints associated with the deletion in cases 1–11 and 18–21 are all located within the REPD region of 8p23.1. The similar deletion segments in cases 1–10 and 18–21 encompass 15–17 OMIM genes, including ARHGEF10, CSMD1, CLN8, DLGAP2, MCPH1, and others. According to the ClinGen database, these genes are not explicit haploinsufficiency genes, and alteration in the dosage change of a single gene is insufficient to cause clinical symptoms. ARHGEF10 is a member of the Rho guanine nucleotide exchange factors (GEFs) family. The gene CUB and Sushi Multiple Domains 1 (CSMD1) is responsible for encoding a sizeable membrane-bound protein that contains a solitary transmembrane domain. Both ARHGEF10 and CSMD1 are implicated in the development of the central nervous system [33, 34]. CLN8 serves as an endoplasmic reticulum cargo receptor and is integral in regulating lysosome formation. DLGAP2, a critical component of the postsynaptic density, regulates synapse-related functions and may play a crucial role in synaptic organization and neuronal signaling. Notably, CLN8 and DLGAP2 are associated with autism, epilepsy and intellectual disabilities [35, 36]. MCPH1 regulates the DNA-damage response and is associated with microcephaly and neurological function [37]. Some researchers propose that DLGAP2, MCPH1 and NEF3 are the candidate genes for autism [38]. Furthermore, literature and the DECIPHER database indicate that patients with 8p23.1-pter deletion show symptoms such as intellectual disability, microcephaly, autism, attention deficit, hyperactivity and speech disorders [8]. In cases 1–10 and 18–21, the sizes of the deletions were relatively consistent, ranging from 6.7 to 7.5 Mb, while case 11 had a deletion of only 4.5 Mb. No significant abnormalities were detected in the fetal ultrasound examination of case 11, and the abnormal chromosome 8 is inherited from her father, who has a normal phenotype. The 2.4 Mb segment at the telomere of 8p (ranging from 158.05 kb to 2.57 Mb) was not deleted in case 11 and may represent a crucial region for inv dup del (8p). This segment contains the OMIM genes DLGAP2, CLN8, and ARHGEF10, which are considered candidate genes for developmental delay, intellectual disability and neurobehavioral disorders. As individual gene haploinsufficiency does not fully explain clinical phenotypes, it is likely that these genes act synergistically, such that combined deletion may affect neurodevelopment [36]. Catusi et al.. also considered that these genes-DLGAP2, CLN8, and ARHGEF10-are candidate genes for the 8p23.2-pter microdeletion syndrome. These genes are believed to contribute to the diverse clinical phenotypes associated with 8p23.2-pter microdeletions [33].

Inv dup del (8p) can result in duplications of varying lengths. In our study, the distal breakpoints of the 8p duplications observed in cases 1–11 and 18–21 correspond to the REPP of the 8p23.1 region, while the proximal breakpoints vary in location. The lengths of the duplications range in length from 8.2 MB to 31.5 Mb. The shortest duplicated segment with a length of 8.2 Mb comprises 27 OMIM genes. In contrast, the longest duplicated segment, spanning 31.5 Mb, contains 175 OMIM genes. Genes such as NRG1 and FGFR1 are known to be involved in brain development. Their abnormal expression or dysfunction may lead to neurodevelopmental disorders [39]. The duplication covers the region from REPP to the centromere but excludes the 8p23.1 region. According to the ClinGen database, these genes are not clearly defined as triplosensitivity. However, these genetic alterations might influence neural development through a complex network of interactions, specific signaling pathways, or modifications induced by other genetic elements or environmental factors. Consequently, the inv dup del(8p) syndrome presents diverse clinical manifestations [40].

In case 12, the 1.8 Mb deletion encompasses a portion of the DLGAP2 gene, but doesn’t include the CLN8 and ARHGEF10 genes. Conversely, the 33.0 Mb duplication segment incorporates the 8p23.1 region. In case 17, the 11.3 Mb deletion involves the DLGAP2, CLN8, ARHGEF10 OMIM genes and the 8p23.1 region. The 8p23.1 region, which contains the GATA4 and SOX7 genes, is recognized as the intermediate neutral copy region in the classic inv dup (8p) configuration. This region is closely associated with the 8p32.1 deletion/duplication syndrome, which differs from the inv dup(8p) syndrome. The breakpoints of the 8p23.1 deletion/duplication are located at two low copy repeats (LCRs): REPD and REPP. This syndrome is characterized by a diverse range of phenotypic manifestations, including congenital heart defects, diaphragmatic hernia, craniofacial anomalies, microcephaly, and developmental delay and/or learning difficulties [41]– [42]. The GATA-binding protein 4 (GATA4) and SOX7 are among the best-known genes associated with heart defects [20, 43]. We hypothesize that the 8p23.1 region is a candidate region for certain inv dup del (8p) cases, such as case 12 and case 17, in which chromosomal variations involve this region. These cases are postulated to be induced by the U - type exchange mechanism.

Differences in the size of the duplicated segments lead to the variability observed in the clinical manifestation of fetuses with inv dup del (8p). In our case study, as well as in previously reported instances of prenatal diagnoses, the severity of the clinical presentation correlates with the length of the duplication segments. Case 5 exhibited a 17.2 Mb duplication, resulting in less severe clinical manifestations, characterized by thick NT and echogenic intracardiac focus. Case 11, which had the smallest duplication of 8.2 Mb, displayed no obvious abnormalities on fetal ultrasound. In other cases where the duplication exceeded 23 Mb, clinical manifestations included brain abnormalities, heart defects, polycystic kidney disease, umbilical hernia, developmental delay, etc. In this study, six fetuses presented with brain abnormalities, including lateral ventricle enlargement, agenesis of the corpus callosum, hypoplasia of the cerebellar vermis, among others. Additionally, two fetuses presented with cardiac defects, such as persistent left superior vena cava, tricuspid regurgitation, and ventricular septal defect. According to chromosome G-banding and CMA, the duplicated regions in 8p23-p11 in previously reported cases exceeded 23 Mb, except for one case with an ambiguous duplication breakpoint. The clinical manifestations in these 9 previously reported cases were relatively severe. Importantly, in fetuses diagnosed with inv dup del (8p), the ultrasound findings predominantly indicated CNS abnormalities (6 out of 9) and congenital heart defects (6 out of 9), suggesting a relatively high prevalence of brain and heart abnormalities associated with inv dup del (8p) chromosomal rearrangements.

The literature concerning infant or postpartum cases indicates a consistent correlation between the length of the duplicated segments and clinical manifestations. Specifically, longer duplicated segments are associated with severe clinical manifestations, including facial, cerebral, and limb malformations, along with severe intellectual disability and autism [2, 10].

During the early to middle stages of pregnancy, several cases of fetal inv dup del (8p) chromosome abnormality were observed without evident ultrasonic structural anomalies. For example, case 5 (gestational age 17 + weeks) presented with fetal ventricular echogenic foci, while case 11 (gestational age 18 + weeks) exhibited mild dilation of the left lateral ventricle, case 19 (gestational age 12 weeks) showed increased nuchal translucency and case 21 (gestational age 11 + 4 weeks) presented with diffuse lymphedema respectively. These findings are commonly recognized as ultrasound soft marker abnormalities and are not specific to inv dup del (8p) syndrome. In some cases, the diagnosis of fetal inv dup del (8p) were unexpectedly diagnosed during prenatal assessments due to serum screening. For instance, cases 5 and 9 were identified based on anomalies detected in serum screening. In case 11, the chromosome 8 abnormality was first detected via non-invasive prenatal screening and subsequently confirmed by CMA as inv dup del (8p) rearrangement. Overall, the majority of ultrasound findings are not specific for inv dup del (8p) syndrome, indicating that further karyotype and CMA analysis is required for accurate diagnosis. For complex chromosomal karyotype abnormalities, it is advisable to conduct CMA to identify potential chromosomal CNVs. If CNVs are present, the breakpoints of the aberrant chromosomes, the size of the deleted or duplicated segments and the genes they contain can be identified, thus facilitating improved genetic counseling. Therefore, for all patients undergoing prenatal diagnosis, we recommend CMA as the preferred diagnostic method. Additionally, when CMA yields complex results that require further clarification, chromosome karyotype analysis should be performed to provide a more comprehensive evaluation.

As fetus development is a dynamic process influenced by multiple factors. In future work, we will expand the sample size, observe a broader range of clinical manifestations, and avoid result biases caused by sample limitations. Conducting full-process fetal imaging assessments will enhance our understanding of fetal development. We aim to validate the current results and improve generalizability of our conclusions.

## Conclusion

Based on the differences in the mechanism of inv dup del (8p) formation, we categorized cases in this study, along with previously reported cases, into two groups. Through an analysis of these cases and literature review, we hypothesize that the 2.4 Mb telomeric region of 8p and the 8p23.1 region may serve as candidate pathogenic regions for this syndrome. Specifically, the genes DLGAP2, CLN8, and ARHGEF10 are key genes in the 2.4 Mb telomeric region of 8p, while GATA4 and SOX7 are key genes in the 8p23.1 region. However, despite the established correlation between the length of the duplicated segment and clinical severity, no critical candidate region has been identified within the duplicated region of 8p22-p11.1 in cases of Inv dup del (8p).

In inv dup del (8p) syndrome, prenatal ultrasound findings are varied, with cerebral and cardiac anomalies being the most common intrauterine phenotypes. Additionally sonographic features may include increased NT, renal anomalies and facial dysmorphism. These findings could provide important clues for the potential prenatal diagnosis of inv dup del (8p) syndrome. However, it should be noted that affected fetuses may not exhibit obvious ultrasound abnormalities during early gestation. CMA enables precise characterization of the genomic rearrangements and copy number variations associated with inv dup del (8p) syndrome. Accurate delineation of the size and localization of genomic alterations helps to clarify the mechanism behind chromosomal rearrangement formation. Therefore, we recommend CMA as the preferred diagnosis method for suspected cases of inv dup del (8p) syndrome.

## Supplementary Information

Below is the link to the electronic supplementary material.

Supplementary Material 1

## Data Availability

The data that support the findings of this study are not publicly available due to individual privacy but are available from the corresponding author (Weiwei Huang, E-mail: huangw12345@126.com) upon reasonable request.

## Abbreviations

AF: Amniotic fluid
AO: Aorta
CB: Cord blood
CHD: Congenital heart disease
CMA: Chromosomal microarray analysis
CNS: Central nervous system
CNVs: Copy number variations
CSMD1: CUB and Sushi Multiple Domains 1
CVS: Chorionic villus sample
GATA4: GATA-binding protein 4
GEFs: Guanine nucleotide exchange factors
LCRs: Low copy repeats
NAHR: Non-allelic homologous recombination
NIPS: Non-invasive prenatal screening
NT: Nuchal Translucency
REPD: Repeat-distal
REPP: Repeat-proximal
RV: Right ventricle
