# Supplementary material for: Prenatal diagnosis and molecular cytogenetic characterization of 12 cases of chromosome 8 inverted duplication deletion syndrome
Source: Orphanet J Rare Dis. 2025 Aug 11;20:421. doi: 10.1186/s13023-025-03969-w (PMC12341122; doi:10.1186/s13023-025-03969-w)
Supplement: Supplementary file 1 — Supplementary Material 1 [file 13023_2025_3969_MOESM1_ESM.pdf]

Certificate of Elsevier Language Editing Services

The following article was edited by Elsevier Language Editing Services:

Prenatal Diagnosis and Molecular Cytogenetic Characterization of 12 Cases of Chromosome 8 Inverted Duplication Deletion Syndrome

Ordered by:

Xi Yang

Estimated Delivery date:

2025-05-16

Order reference:

ASLEEX1104117
